# Cardiac Rehabilitation in a Patient With Severe Heart Failure and Ventricular Septal Defect Secondary to Acute Myocardial Infarction

**DOI:** 10.7759/cureus.19901

**Published:** 2021-11-25

**Authors:** Guillermo A Mazzucco, Leonardo Pilon, Juan Pablo Escalante, Nicolas Chichizola, Rodrigo Torres-Castro

**Affiliations:** 1 Cardiopulmonary Rehabilitation Unit, Instituto Cardiovascular de Rosario, Rosario, ARG; 2 Unidad de Investigación en Kinesiología Cardiorespiratoria, Universidad del Gran Rosario, Rosario, ARG; 3 Cardiology, Instituto Cardiovascular de Rosario, Rosario, ARG; 4 PhysioEvidence, International Physiotherapy Research Network, Barcelona, ESP; 5 Departamento de Kinesiología, Facultad de Medicina, Universidad de Chile, Santiago, CHL

**Keywords:** heart failure, center-based cardiac rehab, heart & lung transplant, exercise training, physical training

## Abstract

The treatment of choice for patients with advanced heart failure (HF) and with limiting symptoms with evidence of a poor prognosis despite optimal conventional treatment is a heart transplant. However, there is little literature dealing with the effects of cardiovascular prehabilitation with an important change in physical capacity, which can influence the admission on the waiting list for a heart transplant. We presented one young male, smoker, with no prior history of cardiovascular disease, severe ventricular dysfunction, interventricular defect, and HF. It was decided to implant a cardioverter-defibrillator as primary prevention of sudden death and start the pre-cardiac transplant evaluation and subsequent inclusion in the waiting list on an elective basis. While waiting for the transplant, cardiopulmonary rehabilitation (CPR) was indicated. After 15 months of CPR, the patient improved his left ventricular ejection fraction (LVEF; 20% to 40%), systolic pulmonary artery pressure (55 to 40 mmHg), and peak oxygen uptake (23.9 to 29.1 ml/kg/min). In this patient, a program of CPR improved cardiac function and physical capacity, allowing him to be removed from the national waiting list for a heart transplant.

## Introduction

The treatment of choice for patients with advanced heart failure (HF) and with limiting symptoms with evidence of a poor prognosis despite optimal conventional treatment is heart transplant [1]. Unfortunately, the limited number of donors available restricts this treatment to a small fraction of potential recipients. Generally, a heart transplant is considered when it is probable to improve quality of life and increase survival [1].

Through the cardiopulmonary exercise testing (CPET), the objective assessment of physical capacity provides prognostic information. In patients, achieving a peak oxygen uptake (VO_2_peak) of <10 mL/kg/min is a strong predictor of poor prognosis [2]. Additionally, in patients under 50 years and women, a predicted VO_2_peak lower than 50% can also be used to guide transplant candidacy in conjunction with absolute value VO_2_peak [1]. Poor aerobic capacity prior to graft deterioration is not only limited to the HF but also caused by peripheral factors, such as limited function in the skeletal muscles and in the blood vessel walls [3].

Exercise training led to an increase in multiple systemic, angiogenetic, and platelet-derived inflammatory mediators [4]. Additionally, exercise activates blood platelets, potentially reflecting increased catecholamines and shear stress, regulates the growth and repair of blood vessels, and promotes nitric oxide production from activated endothelial cells [5-7].

The literature suggests a multifactorial beneficial effect of cardiopulmonary rehabilitation (CPR) after a heart transplant, improving autonomic control, muscle strength, and body composition [8-10], but the evidence is lower previous to a heart transplant, commonly called prehabilitation. Prehabilitation is the practice of enhancing a patient’s functional capacity before surgery, with the aim of improving postoperative outcomes by acting in three aspects: physical activity, adequate nutrition, and reduction of the anxiety and frustration component [11].

Prehabilitation programs have shown efficacy in increasing functional capacity and preventing postoperative complications in some high-risk surgical populations [12]. Nevertheless, patients on the waiting list for heart transplants are commonly considered ineligible for these programs because there is a general opinion that they are too sick to exercise. However, a recent study showed that a prehabilitation program in patients listed for a heart transplant is feasible, safe, and may prevent the deterioration in exercise, functional capacity, and quality of life while waiting for a transplant [13].

In this context, there is little literature dealing with the effects of cardiovascular prehabilitation with an important change in physical capacity, which can influence the admission on the waiting list for a heart transplant.

## Case presentation

A 42-year-old male, office employee, smoker, height 1.66 m, weight 66 kg, with no family history or antecedents of cardiovascular disease, who consulted at Instituto Cardiovascular de Rosario (ICR) for a condition characterized by progressive dyspnea lasting approximately two months, associated with episodes of mid-thoracic pain with radiation to the shoulders two hours long.

**Figure 1:**
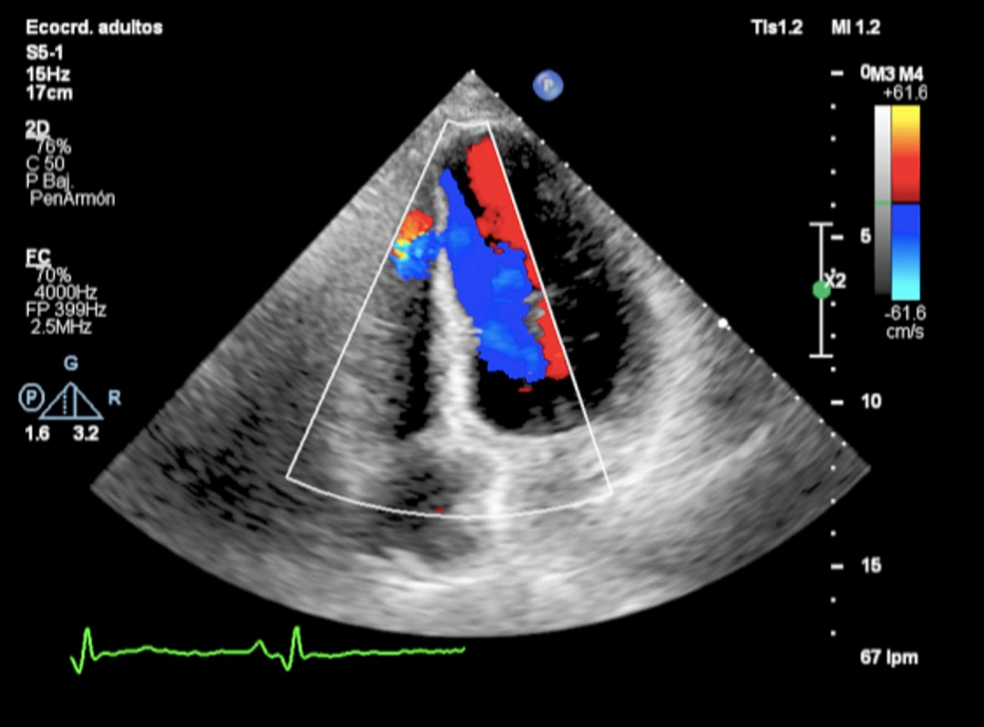
Ventricular septal defect observed by cardiac Doppler echo

In the initial evaluation, signs and symptoms compatible with HF and holosystolic murmur in a polifocal bar were evidenced, with the presence of thrill and gallop rhythm. He was admitted and further evaluated by: (i) routine biochemistry examination within normal parameters except for an elevated N-terminal pro-brain natriuretic peptide (NT-ProBNP) of 5321 pg/ml. (ii) 12-lead electrocardiogram revealed sinus tachycardia of 130 beats per minute associated with signs of inferior-lateral myocardial fibrosis with compromised right leads. (iii) Chest X-ray, where signs of grade III venulo-capillary hypertension were observed. (iv) Color Doppler echocardiogram: systolic and diastolic diameter of the left ventricle 61/55 mm, respectively, impaired left ventricular ejection fraction (LVEF; 20%), abnormal segmental motility with apical akinesia in all its segments, inferior, septal, and anteromedial. Systolic displacement of the plane of the tricuspid annulus was 13 mm and grade II diastolic dysfunction. Pulmonary artery systolic pressure was 55 mmHg. At the junction of the middle and apical third of the interventricular septum, a continuity solution with a left to right flow of approximately 5.5 mm was observed (Table 1 and Figure 1).

**Table 1:** Comparison between pre and post-CPR echocardiograms CPR: cardiopulmonary rehabilitation; LVEF: left ventricular ejection fraction; LVDD: left ventricle diastolic diameter; LVSD: left ventricular systolic diameter; IVC: interventricular communication; SPAP: systolic pulmonary artery pressure; SDTAP: systolic displacement of the tricuspid annulus plane.

	December 26, 2019	May 27, 2021
LVEF (%)	20	40
LVDD (mm)	61	54
LVSD (mm)	55	41
Akinetic cardiac segments (no)	7	3
IVC (mm)	5.5	5.2
Maximum speed CIV (m/sec)	3.9	4.4
SPAP (mmHg)	55	40
SDTAP (mm)	13	22

The condition was interpreted as a mechanical complication of acute inferolateral myocardial infarction with a compromise of the right ventricle not reperfused because it was outside the therapeutic window associated with decompensated HF. He underwent aspirin 100 mg daily (8 hours), clopidogrel 75 mg daily (8 hours), bisoprolol 2.5 mg every 12 hours (8-20 hours), ivabradine 7.5 mg every 12 hours (8-20 hours), enalapril 5 mg every 12 hours (8-20 hours), eplerenone 50 mg per day (8 hours), furosemide 20 mg per day (8 hours), atorvastatin 80 mg per day (20 hours) with good clinical response.

Once his admission condition had stabilized, post-infarction risk stratification was continued by: (i) Cinecoronariography: the left main coronary artery did not present lesions, while angiographically significant stenotic lesions were evidenced (>60%) in the circumflex, anterior descending, and right coronary artery. (ii) Cardiac magnetic resonance imaging (MRI): severely increased ventricular volumes and severely depressed LVEF (28%). At the septoapical level, a 3.5 mm muscular interventricular communication was visualized. Absence of myocardial viability.

Due to the high risk of the patient due to severe ventricular dysfunction, interventricular defect, and HF, it was decided to implant a cardioverter-defibrillator as primary prevention of sudden death and to start the pre-cardiac transplant evaluation and subsequent inclusion on the waiting list on an elective basis. At the time of his discharge, outpatient CPR was indicated.

On January 31, 2020, the patient entered the CPR program. The sessions lasted 60 minutes, where the first 30 minutes were for aerobic training and the remaining 30 minutes for exercises for muscle strength, balance, coordination, and flexibility. ECG telemetric control and pulse oximetry were used to monitor the patient during the first sessions. The patient had a functional class III on the New York Heart Association (NYHA) scale at admission to the program. The first five weeks were for adaptation to training, where the intensity was lower than 4 (0-10) on the Borg scale.

The first cardiopulmonary exercise test (CPET) was performed on a treadmill on March 6, 2020. The protocol used was modified Bruce, where the intensity was increasing every three minutes. The reason for stopping the test was due to exhaustion and the request of the patient in stage 4 of Bruce protocol. At that time, the patient reached a VO_2_peak of 23.9 mL/kg/min, finding his anaerobic threshold at 100 beats per minute (bpm) and 70% of the VO_2_peak reached, and a maximum heart rate toward the end of the test of 124 bpm. Concerning the theoretical maximum heart rate (TMHR), it reached 70%, although the patient was on beta-blocker medication. The hemodynamic response of the patient during the test was normal, without presenting chest pain. The respiratory exchange ratio (RER) was 1.09 (Table 2).

**Table 2:** Comparison between cardiopulmonary exercise tests before and after CPR bpm: beats per minute; CPR: cardiopulmonary rehabilitation; METs: metabolic equivalents; mmHg: millimeters of mercury; VO_2_: oxygen consumption; RER: respiratory exchange ratio.

	January 31, 2020	May 14, 2021
Protocol	Bruce	Bruce
METs	11.7	11.7
Maximum heart rate (bpm)	124	136
Maximum systolic blood pressure (mmHg)	150	160
Maximum diastolic blood pressure (mmHg)	80	80
VO_2_peak (mL/kg/min)	23.9	29.1
Anaerobic threshold (VO_2_%)	70	86
Double product (mmHg x bpm)	18,600	23,200
RER	1.09	1.09

With these results, starting at week 5, the treadmill aerobic training was planned at an intensity of 80% with a variable continuous training method, where progressively, and depending on the patient's responses during the sessions, we modified the training protocol to perform four minutes at 80% interspersed by two minutes of recovery at 40%. Concerning the strength exercises, 4 or 5 exercises were performed per session, with an intensity measured by the Borg scale that should not be higher than level 5 when reaching the last repetitions of each series. As in aerobic training, depending on the patient's responses and adaptations, the number of series, number of repetitions, and complexity of the exercises progressed with the running of the sessions. The training protocol was carried out based on the recommendations of the American Association of Cardiovascular and Pulmonary Rehabilitation, the American Heart Association, and the American College of Cardiology guidelines [14].

After 15 months within the CPR program and 122 sessions carried out, with a frequency of two-weekly sessions, a new follow-up CPET with the same protocol was carried out on May 14, 2021. It is essential to clarify that the patient was with the same medication as the baseline, although, at that time, he had NYHA functional class 0 and gained 3 kg. The test was stopped in stage 4 at the request of the patient. At the end of the test, the patient reached a VO_2_peak of 29.1 mL/kg/min, finding his anaerobic threshold at 125 bpm and 86% of VO_2_peak and a maximum heart rate of 136 bpm (82% of his TMHR). The RER was 1.09.

A second color Doppler echocardiogram was performed in May 2021, finding: left ventricular systolic and diastolic diameter of 54/41 mm, respectively, LVEF with moderate deterioration of 40%, abnormal segmental motility with apical, inferior and inferoseptal akinesia. Systolic displacement of the plane of the tricuspid annulus of 22 mm. Pulmonary artery systolic pressure of 40 mmHg. Interventricular communication of approximately 5.2 mm.

Due to the clinical improvements of the patient, the VO_2_peak value was reached during the second CPET and the absence of hospitalizations was associated with HF in this period, his cardiologist decided to exclude him from the national waiting list for a heart transplant and to continue within the CPR program, to maintain and/or improve his current physical condition.

## Discussion

HF harms the health-related quality of life (HRQoL), both in physical function and psychosocial function, worsening the deterioration with increased functional class [15]. In this case, we can see how the benefits of CPR make a person leave the waiting list for a heart transplant.

CPR has been widely demonstrated to improve physical fitness, functional capacity, and HRQoL in chronic HF patients [16]. Even more, recent meta-analyses showed that exercise-based CPR improved HRQoL and exercise capacity in patients with HF [17]. However, the evidence is less in patients who will have to undergo cardiac transplants and focuses almost exclusively on post-transplant interventions [18].

In this case, the exercise was before the transplant, a concept defined as prehabilitation. A recent study evaluated a multimodal prehabilitation intervention in HF patients awaiting heart transplant showing that CPR is feasible, safe and may prevent the deterioration in exercise, functional capacity, and quality of life while waiting for a transplant [14].

The VO_2_peak is considered the best predictor of survival in cardiovascular diseases [19]. CPET precisely defines maximum exercise capacity through the measurement of VO_2_peak. In addition, VO_2_peak values have a critical role in informing patient selection for advanced HF interventions such as heart transplants [20]. 

Previous studies have indicated that a peak aerobic power ≤10 mL/kg/min is a strong predictor of a poor prognosis in patients with HF [21]. In addition, deterioration of functional class and low oxygen consumption levels are variables that indicate a heart transplant [22]. Thus, CPR plays a fundamental role in improving both so that patients do not reach the transplant or, if they do, that they can cope with it in a better way [17].

In this present case, the exercise capacity analyzed through the measurement of VO_2_peak improved from 23.9 to 29.1 ml/kg/min, and more important was the improvement in the appearance of the anaerobic threshold, which would suggest that the patient was less fatigued and more resistant to the aerobic effort, due to the rightward deviation of the threshold. One possible explanation is physiological and clinical adaptations to training, particularly peripheral muscle adaptations that contribute to the improvements shown in the CPET [23].

Further studies will be needed to show the effects of the prehabilitation in this population and to suggest a CPR guideline in patients with severe HF awaiting a heart transplant.

## Conclusions

As shown in this case report, the authors were able to carry out safe and effective CPR, which allowed the patient to significantly improve their quality of life and exercise capacity and get off the cardiac transplant list for clinical stability. Therefore, CPR can be a good strategy in patients who are candidates for a heart transplant, particularly in many developing countries, where referral of patients to a heart transplant list for presurgical CPR is not frequent.
